# Anti-inflammatory and Immunomodulatory Potency of Selenium-Enriched Probiotic Mutants in Mice with Induced Ulcerative Colitis

**DOI:** 10.1007/s12011-022-03154-1

**Published:** 2022-02-21

**Authors:** Abd El-Nasser Khattab, Ahmed M. Darwish, Sarah I. Othman, Ahmed A. Allam, Haifa A. Alqhtani

**Affiliations:** 1 Genetics and Cytology Department, Biotechnology Research Institute, National Research Centre, Dokki, 12622, Giza, Egypt; 2 Cell Biology Department, Biotechnology Research Institute, National Research Centre, Dokki, 12622, Giza, Egypt; 3 Department of Biology, Faculty of Science, Princess Nourah bint Abdulrahman University, P.O. BOX 84428, Riyadh, 11671 Saudi Arabia; 4 Department of Zoology, Faculty of Science, Beni-Suef University, Beni Suef, 65211 Egypt

**Keywords:** Anti-inflammatory, Selenium, Probiotic mutant, Ulcerative colitis, Mice

## Abstract

Selenium-enriched *Lactobacillus plantarum* and *Bifidobacterium longum* mutants were used as a protector against Piroxicam-induced ulcerative colitis (UC). In this study, 32 BALB/c male mice were distributed to four groups: the control group, the Piroxicam group which was given 0.8 mg Piroxicam, SP and SB groups which were given 0.8 mg Piroxicam, and plus *Lactobacillus plantarum* and *Bifidobacterium longum* selenium-enriched mutants, respectively. Bodyweight; serum content of IgG, IgM, TNF-α, IL-2, IL-6, and IL-10; CBC; myeloperoxidase enzyme activity; histopathological examination of colon and spleen; and expression of TNF-α, IL-2, IL-6, and IL-10 genes in colon and spleen with qRT-PCR were determined. Bodyweight was found to reduce in the Piroxicam group and then recovery in the SB group. Serum content of IgG, IL-2, and IL-10 reduced in the Piroxicam group, whereas IgG, TNF-α, and IL-6 increased in the Piroxicam group in comparison to the other groups. Myeloperoxidase activity witnessed a significant increase in the Piroxicam group compared with the other groups. No significant differences were observed between all groups in measurements of red cells, hemoglobin, neutrophil, monocyte, eosinophil, and basophil in blood. Meanwhile, the white blood cells and platelets recorded the highest and lowest value, respectively, in the Piroxicam group. The colon of the Piroxicam group showed a noticeably massive infiltration of inflammatory cells in the lamina propria. These inflammations were mildly reduced in the SP group, while the reduction in the SB group was significant. In the Piroxicam group, splenic parenchyma saw an increase in the number of melanomacrophages, while hypertrophic plasma cells were observed in the SP group. The spleen of the SB group exhibits a nearly normal form. TNF-α and IL-6 genes had significantly upregulated in the colon of the Piroxicam group compared to the control group, while they were significantly downregulated in the SB group. In contrast, IL-2 and IL-10 genes had upregulated in the colon of the SB group compared to the control groups, while they had downregulated in the Piroxicam group. The expression of these genes had not recorded significant differences between all groups in the spleen. Therefore, this study recommends *Bifidobacterium longum* selenium-enriched mutants as anti-inflammatory and immunomodulatory supplements.

## Introduction

As a vital trace element for both animal and human health, selenium (Se) is found in various selenoproteins that perform important biological activities [1]. Since selenium is incorporated in various enzymes and proteins, it also serves as an enzymatic and stabilizing agent. In addition to preventing vascular diseases, selenium boosts the immune system and has antiproliferative properties. Selenium helps to keep thyroid function in balance [2]. Several enzymes, particularly those with antioxidant activity, include selenium, such as glutathione peroxidase (GPx), iodothyronine deiodinase (DIO), and thioredoxin reductase (TRxR). These enzymes inhibit hydrogen peroxide generation via the phospholipid membrane of cells [1]. Selenium allows living organisms to maintain proper physiological activities [3]. Lactic acid bacteria (LAB) and bifidobacteria are significant food-grade bacteria with several functions such as antimicrobial activity, antioxidant activity, vitamins creation, and exopolysaccharides (EPS) biosynthesis, which are all fundamental technical and functional aspects of fermented foods. In addition, the capacity to bind, absorb, and biotransform metal ions from the media into the organic form is one characteristic documented in LAB. Se administration has also been effective in the treatment of inflammation, cancer, cardiovascular disease, immunological responses, male fertility, and thyroiditis [4].

Probiotic microorganisms taken orally can potentially improve the health of consumers. Probiotics are linked to several health benefits involving cholesterol reduction, immune system activation, the reduction of inflammatory bowel disease (IBD), and allergies [5, 6]. According to Gordon and Pesti [7], the gut microbiota has a significant impact on the host’s immunology, biochemistry, physiology, and nonspecific disease resistance. These findings have led to the hypothesis that health could be improved by altering the composition of the gut microbiota through dietary supplements. Probiotic strains of the *Bifidobacterium* and *Lactobacillus* genera that have received both qualified presumption of safety (QPS) and generally recognized as safe (GRAS) approvals are widely employed in the food industry [8]. To expand the range of healthy milk products, additional bioprospecting is needed to find new non-bovine LAB strains with good probiotic qualities. *Lactobacillus* and *Bifidobacterium* are two of the most regularly utilized probiotic microorganisms [9]. Lactobacilli are Gram-positive rods that do not form spores, produce catalase, nor reduce nitrate, in addition to being usually nonmotile. *Lb. acidophilus*, *Lb. salivarius*, *Lb. casei*, *Lb. plantarum*, *Lb. fermentum*, and *Lb. brevis* are the most commonly used lactobacilli species [10]. Bifidobacterium, on the other hand, are Gram-positive rods that do not generate spores and have distinct cellular bifurcating or club-shaped morphologies. *B. animalis*, *B. longum*, *B. bifidum*, and *B. infantis* are the most commonly used species.

The most researched probiotic LAB, which belongs to the *Lactobacillus* and *Bifidobacterium* genera, has been regularly employed in health promotion for both humans and animals, in addition to being used in biological therapies [11]. Ulcerative colitis is described as the loss of epithelial barrier integrity, mucosal and submucosal inflammation, and dysregulated immune responses. Treatment of moderate and severe ulcerative colitis has been conducted by reducing the inflammatory cascade, such as pro-inflammatory cytokines (tumor necrosis factor and interleukin) [12]. TNF-α is one of the major pro-inflammatory cytokines that induce strong inflammatory processes in patients with inflammatory bowel diseases [13]. It is included in the pathogenesis of many diseases, including diabetes, sepsis, rheumatoid arthritis, cancer, Crohn’s disease, and ulcerative colitis [14]. IL-10 and TGF-β play an immunoregulatory role [15]. IL-2 deficiency in mice causes the development of infections intestinal disease similar to ulcerative colitis in humans [16]. Recently, the immunomodulatory effects of probiotics have been evaluated. Dietary probiotics could be used as feed supplements to support the immune system and promote young ducks health, due to their ability to regulate the gene expression of pro-inflammatory cytokines, such as IL-6 [17]. A mixture of *Lactobacillus* and *Bifidobacterium* species causes the upregulation of IL-10 gene and the downregulation of TNF-α and IL-6 genes, as well as the significant increase in levels of IgA and IgG in rats [18].

There have been numerous studies on the use of probiotics as an anti-inflammatory, immunomodulatory, anti-obesity, and other applications. Yet, there is a paucity of literature and studies on the use of probiotics that belong to the *Lactobacillus* and *Bifidobacterium* genera, which are supplemented with important elements for public health, such as selenium. Thus, the aim of this study is to discover novel selenium-enriched *Lactobacillus plantarum* and *Bifidobacterium longum* mutants via EMS mutagenesis. The study evaluates two selenium-enriched mutants of *L. plantarum* and *B. longum* as anti-inflammatory and immunomodulatory probiotics against the Piroxicam medication, in vivo in mice, based on general probiotic criteria, such as tolerance testing of acid, hydrogen peroxide, bile salt, and response to lysozyme and antibiotic, as well as total antioxidant activity.

## Methods

### Bacterial Strains and Growth Conditions of Probiotics

The two wild-type probiotic strains (*Lactobacillus plantarum* Pro1 and *Bifidobacterium longum* ProBl) under the accession numbers MT505334.1 and MZ496550.1, respectively, were obtained from the Applied Microbial Genetics Lab., Genetics and Cytology Dept., the National Research Centre, in Dokki, Cairo, Egypt. The probiotic strains and mutants were cultured in MRS broth (50 mL) at 37 °C for 24 h without shaking.

### EMS Mutagenesis and Selenium-Resistant Mutant Induction

Five milliliters of probiotic strains cultured in MRS for 24 h were centrifuged (6000 rpm, 4 °C, 5 min). The cells were re-suspended in 5 mL of sodium phosphate buffer (pH 7.0, 50 mM), and then EMS (200 mM) was added to the suspension. At 30 °C, tubes were agitated (100 rpm) for 20 and 60 min. Then, 500 µL of sodium thiosulfate (0.4 M) was added to tubes to neutralize the EMS. Cells were centrifuged at (6000 rpm, 4 °C, 5 min) at 4 °C and washed twice with the same buffer, and the cell biomass was re-suspended in phosphate buffer (pH 7.0, 50 mM). The first dilution (10–1) of EMS-treated strain was plated on the surface of MRS supplemented with different concentrations of selenium oxide (10, 20, 30, 40, and 50 ppm). The plates were incubated at 37 °C for 2 days, and selenium-resistant fast-growing colonies were counted.

### High Capacity of Selenium Uptake by Selenium-Resistant Fast-Growing Mutants

After the wild-type probiotic strains and selenium-resistant fast-growing EMS mutants were cultured in MRS broth (50 mL) at 37 °C for 24 h, selenium (IV) oxide (100 ppm) was added. The flasks were then incubated for another 24 h under the same conditions. The selenium uptake was measured for each of the fast growth selenium-resistant EMS mutants and the wild-type strains.

### Determination of Selenium Uptake

Using Variamine Blue (VB) as a chromogenic reagent, Khattab et al. [19] used a rapid and sensitive spectrophotometric approach for the measurement of trace quantities of selenium. An aliquot of a sample solution containing 2 µg of selenium was transferred into a series of 10-mL calibrated tubes. One milliliter of 2% potassium iodide was added, followed by 1 mL of 2 M HCl. The mixture was gently shaken until a yellow color appeared, indicating that the iodine was liberated. Then, 2 mL of a 1 M sodium acetate solution and 0.5 mL of 0.05 percent Variamine Blue (VB) were added. The mixtures were diluted to the desired strength with distilled water and thoroughly blended. At 546 nm, the absorbance of the colored samples was determined and compared to a reagent blank. All absorbance measurements were performed with a Shimadzu UV–VIS spectrophotometer model UV-240 with 1 cm matched quartz cells.

### Quality Assessment of the Mutants and Wild-Type Probiotic Strains

#### Acid Tolerance

The two mutants of probiotics (PSe40/60/1 and BSe50/20/1) and the two wild-type probiotic strains were cultured in MRS broth at 37 °C for 24 h. An aliquot of 0.1 mL from each culture was inoculated into 10 mL MRS broth with pH values adjusted to 2, 3, and 4 and was incubated for 6 h at 37 °C. The cell growth was measured by observing the absorbance at a 600 nm with a UV spectrophotometer (Shimadzu UV–VIS model UV-240).

#### Hydrogen Peroxide Tolerance

The ability of probiotic mutant strains and two wild-type probiotic strains to tolerate hydrogen peroxide was determined using the method of Das and Goyal [20]. *Lactobacillus* strains were cultivated overnight, injected at 1% (v/v) into 100 mL MRS medium (control) and 100 mL MRS medium with 0.75, 1.5, or 2.25 mmol/L hydrogen peroxide, and then incubated at 37 C for 8 h. The cell growth was measured by marking the absorbance at a 600 nm with a UV spectrophotometer.

#### Tolerance to Bile Salts

The overnighted culture of mutant and two wild-type probiotic strains were added to 20 mL of 0.1 M PBS with three different concentrations of bile salts (0.25, 0.50, and 0.75%) and then centrifuged at 4500 rpm for 10 min. The acquired pellet was washed and re-suspended in PBS to achieve a 10^9^ CFU.mL^−1^. The cell growth was measured after 8 h by a UV spectrophotometer at a 600 nm according to Riane et al. [21]

#### Lysozyme Response Assay

The pellet of cells of mutant and two wild-type probiotics strains was re-suspended in 10.0 mL of sterile saline solution with two different concentration of lysozyme (100 and 200 mg L^−1^, Bio Basic Canada INC) and incubated for 3 days at 37 °C according to Pinto et al. [22]. The survival rate was calculated according to the following equation:

$$\mathrm S\mathrm u\mathrm r\mathrm v\mathrm i\mathrm v\mathrm a\mathrm l\;\mathrm r\mathrm a\mathrm t\mathrm e\;\%=\mathrm{Final}(\mathrm{Log}^{10}\mathrm{cfu}/\mathrm{mL})/\mathrm{Initial}(\mathrm{Log}^{10}\mathrm{cfu}/\mathrm{mL})\times100.$$

#### Total Antioxidant Activity (TAA)

This assay was created using the method provided by Riane et al. [21]; 100 mL of ascorbic acid (5 mmol.L^−1^) was combined with 4.9 mL PBS (0.2 mol.L^−1^) at pH 7.0. A 100µL of the tested strain’s pellet was added to the mixture and then incubated at 37 °C for 10 min. The reference was prepared by replacing the sample by 100 µL of PBS. The absorbance was measured at 265 nm by a UV spectrophotometer. The TAA was calculated as the percentage of inhibition of ascorbic acid auto oxidation as follows:

$$\mathrm{TAA }= [ 1 - {\mathrm{A}}_{265} (\mathrm{sample})/{\mathrm{A}}_{265} (\mathrm{reference})] \times 100$$

#### Antibiotic Response of the Wild-Type Probiotics Strains

The disc diffusion method [23] was used to assess antibiotic susceptibility. The two mutants and two wild type of probiotics strains were inoculated on MRS agar (1.0% agar). They were then incubated at 37 °C for 24 h, with an antibiotic disc on top to allow the antibiotics to diffuse into the media. To determine the isolate’s susceptibility, the inhibition zone around each antibiotic disc was measured.

#### Preparation of the Se-Probiotic Supplements

Selenium (IV) oxide (100 ppm) was introduced after the selenium-resistant fast-growing EMS mutants were grown in the identical conditions for 24 h. Under the same conditions, the flasks were incubated for another 24 h. Ten milliliter of bacterial suspension (2.5 × 10^6^/mL) was centrifuged at 6000 rpm for 5 min and suspended with 1000 mL of mice drinking water supplemented, with or without the Piroxicam (200 ppm).

#### Mice Experimental Design

Thirty-two BALB/c male mice were divided into four groups, with 8 mice per cage. Mice weighed 22–25 g at the old age of 2 weeks and were maintained at 22 ± 2 °C with 50% ± 5% humidity and 12 h light/dark cycle. The control group was fed on a normal diet (Table 1) for 6 weeks. The Piroxicam group was given 0.8 mg of Piroxicam/kg body weight daily for 6 weeks, via adding 200 ppm of Piroxicam to 1000 mL of drinking water. The SP group was given 0.8 mg Piroxicam/kg body weight daily during the first week and then given selenium-enriched PSe40/60/1 mutant plus 0.8 mg Piroxicam in 1000 mL of drinking water in the next consecutive 5 weeks. The SB group was given 0.8 mg Piroxicam/kg body weight daily during the first week and then given selenium-enriched BSe50/20/1 mutant plus 0.8 mg Piroxicam in 1000 mL of drinking water in the next consecutive 5 weeks. Mice were individually weighted weekly by electronic balance.

**Table 1:** Diet components of mice

Components	Ratio
Protein	21%
Fat	3.48%
Raw fiber	3.71%
Energy (Kcl/g)	2.95
Yellow corn + soybean meal	44%
Corn gluten	60%
Dicalcium phosphate	
Limestone	
Soybean oil	
Anti-toxin and anti-fungal	
Salt	

### Biochemical Analysis

#### Myeloperoxidase (MPO) Activity

Colon tissues (100 mg) were homogenized in hexadecyltrimethylammonium bromide (0.5%) plus 50 mM of potassium phosphate buffer (pH 6). They were sonicated in an ice bath for 10 s and then cooled three times in ice, with each repeated sonication. The homogenized samples were centrifuged for 10 min at 8000 rpm. 0.1 mL of the sample was mixed with 2.9 mL of 50 mM of phosphate buffer (pH 6.0) containing 0.167 mg/mL o-dianisidine dihydrochloride and 0.0005% hydrogen peroxide. MPO activity was measured with a spectrophotometer at 460 nm for 5 min (Shimadzu model UV-240).

#### Immunological and Cytokines Analysis

The serum content of IgG, IgM IL-2, IL-6, IL-10, and tumor necrosis factor (TNF-α) were determined using specific Elisa kits obtained from Genorise Scientific, Inc.

#### Colon and Spleen Histopathology

Colon and spleen tissue samples were taken from all the mice in the four groups. The samples were fixed in 10% neutral buffered formalin, processed routinely, and stained with hematoxylin and eosin (H&E). Histopathological studies were undertaken through light microscopy, and photomicrographs were made.

#### Gene Expression

Colon (*n* = 32) and spleen (*n* = 32) tissues were individually transferred into 750μL of easy-RED (iNtRoN Biotechnology, Inc., Korea) and homogenized by homogenizer (Labnet, USA). The purity and concentration of RNA were determined by NanoDrop 200c (Thermo Scientific, USA). One microgram of the total RNA was immediately reverse transcribed with iScript cDNA Synthesis Kit (Bio-Rad, USA) and then was stored at − 20 °C until used. The mRNA level of TNF-α, IL2, IL6, and IL10 were determined. In addition, a mRNA relative expression of reference gene, coding β-actin, was used for data normalization. The primer sequences were synthesized by Macrogen Company, Seoul, Korea (Table 2). The cycling conditions included initial denaturation at 94 °C for 3 min, followed by 40 cycles at 93 °C for 45 s, and 60 °C for 15 s. A final extension step was carried on for 5 min at 72 °C. A melting curve was 50–95 °C, with a reading taken at every 0.5 °C, for each individual RTPCR plate. Each sample was subjected to real-time PCR in duplicate. Mean values of duplicates were used for the subsequent analysis. The CT values of the studied genes were normalized to an average of CT value of the reference gene (ΔCT), and the relative expression of each gene was calculated as 2–ΔCT. These expression levels were then used for comparative data analysis.

**Table 2:** Primer sequences

Gene name	Accession number	Sequences (5′ → 3′)	Size
TNF	NM_001278601.1	F- ATCCGCGACGTGGAACTG	70
		R- ACCGCCTGGAGTTCTGGAA	
IL-2	NM_008366.3	F- TCCACTTCAAGCTCTACAG	247
		R- GAGTCAAATCCAGAACATGCC	
IL-6	NM_001314054.1	F- TTCCATCCAGTTGCCTTCTTGG	360
		R- CTTCATGTACTCCAGGTAG	
IL-10	NM_010548.2	F- ACCTGGTAGAAGTGATGCCCCAGGCA	237
		R- CTATGCAGTTGATGAAGATGTCAAA	
β-actin	NM_007393.5	F- GCTCTGGCTCCTAGCACCAT	75
		R- GCCACCGATCCACACAGAGT	

### Statistical Analysis

All observed data were analyzed by one-way ANOVA test, using SPSS program version 18. The analyses of the relative quantification by RT-qPCR were performed using the 2-ΔΔCT value method. The significant differences between means were also calculated using Duncan at *P* < 0.05.

## Results

The main purpose of using EMS mutagen to generate mutations is to create highly genetically efficient mutants that can adsorb large amounts of selenium. After using the Piroxicam drug to induce colitis in mice, these mutants could be introduced into their drinking water as a supplement, in order to test the anti-inflammatory and immunomodulatory effectiveness of the mutants.

### EMS Mutagenesis and Selenium-Resistant Mutant Induction

The ability of a bacterial strain to adsorb significant levels of selenium is known to be proportional to its tolerance to high selenium concentrations. As a result, mutations were introduced as to generate a huge number of genetic variations, and then mutants tolerant to various selenium concentrations were chosen from among them. Varied results were observed when the *Lactobacillus plantarum* Pro1 strain was exposed to the EMS mutagen and then placed on plates with different concentrations of selenium from 10 to 50 ppm, as shown in Table 3. Results demonstrate that at a concentration of 10 ppm of selenium and after a 60-min EMS mutagenic treatment, the largest percentage (28) of selenium-resistant colonies was achieved. In addition, after 20 min of mutational treatment and selection on 10 ppm of selenium, a high percentage (26) was obtained. After mutagenic treatment for 20 min, 16% of selenium-resistant colonies were obtained at a concentration of 20 ppm of selenium. The lowest percentage (4) was obtained after 20 min of mutational treatment and selection on 50 ppm of selenium. After mutagenic treatment, fast-growing colonies were monitored in the presence of high selenium concentrations, in order to select and evaluate their ability to adsorb huge amounts of selenium and convert them from metallic to organic selenium. After mutagenic treatment for 60, 20, and 20 min, three fast-growing colonies were formed in the presence of concentrations 10, 20, and 30 ppm of selenium. Furthermore, diverse effects were obtained when the *Bifidobacterium longum* ProBl strain was exposed to the EMS mutagen and then placed on plates with varied doses of selenium from 10 to 50 ppm, as indicated in Table 3. Results show that at a selenium concentration of 10 ppm and a 20-min EMS mutagenic treatment, the highest percentage of selenium-resistant colonies (38) was obtained. A significant percentage (32) was obtained after 60 min of mutational treatment and selection on 10 ppm selenium. At a selenium concentration of 20 ppm, 24% of selenium-resistant colonies were obtained after a 20-min mutagenic treatment. After 60 min of mutational therapy and selection on 50 ppm selenium, the lowest percentage 8 was obtained. Fast-growing colonies were selected. In the presence of high selenium concentrations after mutagenic treatment, they were evaluated for their ability to absorb large amounts of selenium, and convert them from metallic to organic selenium. Results show that at a selenium concentration of 10 ppm and a 20-min EMS mutagenic treatment, the highest number of fast-growing colonies (4) was obtained. In the presence of concentrations of 10, 20, and 30 ppm of selenium, three fast-growing colonies were established after mutagenic treatment for 60, 20, and 60 min. When evaluating the selenium-resistant fast-growing EMS mutants of *Lactobacillus plantarum*, it was found that the improvement rate in selenium adsorption ranged from 315.98 (mutant PSe40/60/1) to 196.74 (mutant PSe10/20/2), as shown in Table 4. The data confirm that all mutants proved to have a higher selenium biosorption than the untreated original strain did. In addition, when the selenium-resistant fast-growing EMS mutants of *Bifidobacterium longum* were evaluated, it was discovered that majority of the mutants carried more selenium than the original untreated strain did, as shown in Table 4. The greatest mutant was BSe40/60/1, which carried a significant amount of selenium, reaching 91.36 ppm, followed by the BSe50/20/1 mutant, for which the selenium uptake at 241.15% was greater than in the original untreated strain (ProBl).

**Table 3:** Selenium-resistant and selenium-resistant fast-growing colonies obtained after EMS mutagenesis of *Lactobacillus plantarum* Pro1 and *Bifidobacterium longum* ProBl

Selenium oxide conc. (ppm)	Exposure time (min)	No. of tested colonies	No. of resistant colonies		% Of resistant colonies		No. of selenium-resistant fast-growing colonies	
			Pro1	ProB1	Pro1	ProB1	Pro1	ProB1
10	0	100	2*	4*	2	4	0	0
	20	50	13	19	26	38	2	4
	60	50	14	16	28	32	3	3
20	0	100	3*	2*	3	2	0	0
	20	50	8	12	16	24	3	3
	60	50	6	9	12	18	1	2
30	0	100	0	2*	0	2	0	0
	20	50	7	10	14	20	3	1
	60	50	5	7	10	14	1	3
40	0	100	0	0	0	0	0	0
	20	50	5	7	10	14	1	0
	60	50	4	8	8	16	1	2
50	0	100	0	0	0	0	0	0
	20	50	2	6	4	12	1	2
	60	50	3	4	6	8	2	1

^*^Indicates to petite colonies

**Table 4:** Selenium biosorption of selenium-resistant fast-growing EMS mutants of *Lactobacillus plantarum* Pro1 and *Bifidobacterium longum* ProBl grown in the presence of selenium oxide (100 ppm)

Mutant no	Residual se (ppm)	Biosorption-se (ppm)	% to W.T (Pro1)	Mutant no	Residual se (ppm)	Biosorption-se (ppm)	% to W.T (ProBl)
W.T (Pro1)	71.5^a^ ± 0.5	28.5^ g^ ± 0.5	100.00	W.T(proB1) (ProBl)	62.6^a^ ± 0.6	37.4^ g^ ± 0.6	100.00
PSe10/20/1	26.4^d^ ± 0.3	73.6^d^ ± 0.3	257.99	BSe10/20/1	26.6^c^ ± 0.4	73.4^e^ ± 0.4	196.04
PSe10/20/2	43.9^b^ ± 0.4	56.2^f^ ± 0.4	196.74	BSe10/20/2	18.9^d^ ± 0.2	81.1^c^ ± 0.2	216.68
PSe10/60/1	26.7^d^ ± 0.3	73.3^d^ ± 0.3	256.90	BSe10/20/3	21.6^d^ ± 0.3	78.4^d^ ± 0.3	209.57
PSe10/60/2	25.8^d^ ± 0.5	74.2^d^ ± 0.5	260.06	BSe10/20/4	35.6^b^ ± 0.4	64.4^f^ ± 0.4	172.07
PSe10/60/3	18.93^e^ ± 0.2	81.1^bc^ ± 0.2	284.06	BSe10/60/1	17.6^de^ ± 0.2	82.4^c^ ± 0.2	220.12
PSe20/20/1	17.7^ef^ ± 0.3	82.3^b^ ± 0.3	288.23	BSe10/60/2	18.6^d^ ± 0.3	81.4^c^ ± 0.3	217.42
PSe20/20/2	26.9^d^ ± 0.1	73.1^d^ ± 0.1	256.27	BSe10/60/3	24.9^c^ ± 0.2	75.1^e^ ± 0.2	200.80
PSe20/20/3	36.9^c^ ± 0.4	63.1^e^ ± 0.4	220.88	BSe20/20/1	19.6^d^ ± 0.1	80.4^d^ ± 0.1	214.75
PSe20/60/1	22.7^e^ ± 0.2	77.3^c^ ± 0.2	270.71	BSe20/20/2	26.8^c^ ± 0.2	73.2^e^ ± 0.2	195.59
PSe20/60/2	19.4^e^ ± 0.3	80.6^c^ ± 0.3	282.29	BSe20/20/3	14.4^e^ ± 0.4	85.6^b^ ± 0.4	228.81
PSe30/20/1	17.5^ef^ ± 0.2	82.5^b^ ± 0.2	289.21	BSe20/60/1	14.8^e^ ± 0.3	85.2^b^ ± 0.3	227.66
PSe30/20/2	18.7^e^ ± 0.3	81.3^bc^ ± 0.3	284.79	BSe20/60/2	23.6^c^ ± 0.2	76.4^de^ ± 0.2	204.12
PSe30/20/3	27.5^d^ ± 0.1	72.5^d^ ± 0.1	254.20	BSe30/20/1	14.8^e^ ± 0.3	85.2^b^ ± 0.3	227.69
PSe30/60/1	21.7^e^ ± 0.4	78.3^c^ ± 0.4	274.42	BSe30/60/1	21.7^d^ ± 0.1	78.3^d^ ± 0.1	209.38
PSe30/60/2	14.6^f^ ± 0.2	85.4^b^ ± 0.2	299.09	BSe30/60/2	16.5^e^ ± 0.2	83.5^c^ ± 0.2	223.06
PSe40/20/1	16.4^f^ ± 0.3	83.6^b^ ± 0.3	292.99	BSe30/60/3	13.8^e^ ± 0.3	86.2^b^ ± 0.3	230.44
PSe40/60/1	9.8^ g^ ± 0.1	90.2^a^ ± 0.1	315.98	BSe40/60/1	8.6^ fg^ ± 0.2	91.4^a^ ± 0.2	244.15
PSe50/20/1	11.4^ g^ ± 0.2	88.6^a^ ± 0.2	310.55	BSe40/60/2	12.8^f^ ± 0.2	87.2^ab^ ± 0.2	232.95
PSe50/60/1	10.1^ g^ ± 0.3	89.9^a^ ± 0.3	314.93	BSe50/20/1	9.8^f^ ± 0.1	90.2^a^ ± 0.1	241.15
PSe50/60/2	12.9^ fg^ ± 0.3	87.1^ab^ ± 0.3	304.94	BSe50/20/2	12.9^f^ ± 0.1	87.1^b^ ± 0.1	232.87
				BSe50/60/1	11.9^f^ ± 0.2	88.1^a^ ± 0.2	235.44
*P*-value	0.03	0.03			0.04	0.04	

### Quality Evaluation of the Two Mutants and Wild Types of Probiotic Strains

#### Acid Tolerance

**Fig. 1:**
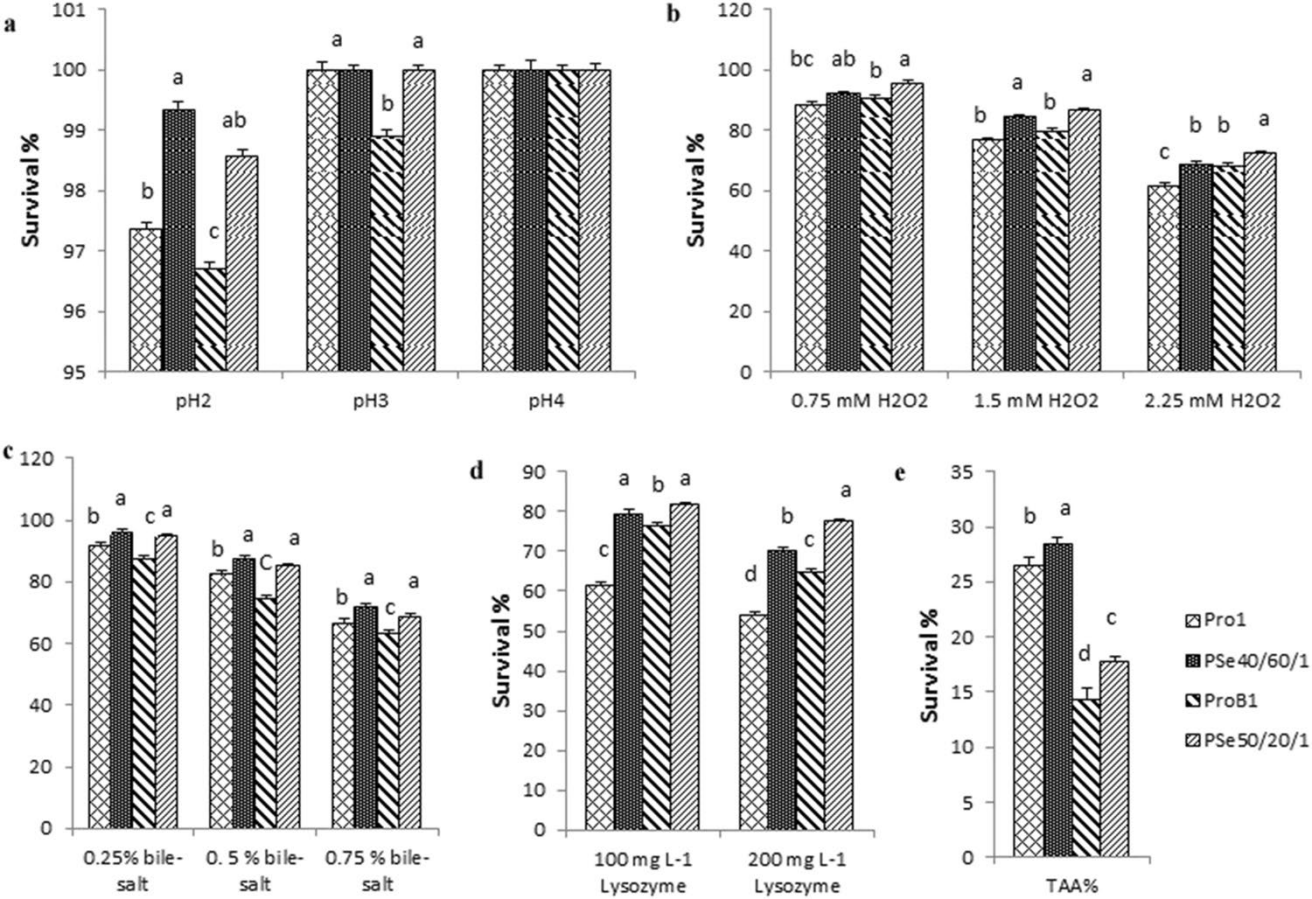
**a** Show the survival percentages of the two mutants and wild kinds of probiotics strains after 6 h at pH 2, 3, and 4, **b** show survival % of the two mutants and wild types of probiotics strains under different concentrations of H_2_O_2_ after 8 h, **c** show survival % of the two mutants and wild types of probiotics strains under different concentrations of bile salts after 8 h, **d** show survival % of the two mutants and wild types of probiotics strains under different concentrations of lysozyme after 2 h, and **e** show TAA % of the two mutants and wild types of probiotics strains

The difference in viable cell counts after incubation for 0 min and 6 h was used to assess the survival rate of chosen isolates in acidic buffer, as indicated in Fig. 1a. All the strains showed better tolerance to acidic pH of 2.0, when compared to normal conditions. Mutant (PSe40/60/1) showed a high survival rate at pH 2.0 and 99.35% survival rate. In general, the mutants were more able to tolerate the acidic medium than the parental strains did. The PSe40/60/1 mutant had a higher tolerance for acid conditions, compared with the BSe50/20/1mutant.

#### Hydrogen Peroxide Tolerance

The difference in viable cell counts was used to assess the survival rate of the selected strains after 8 h of incubation, as shown in Fig. 1b. Under 0.75 mM H_2_O_2_, all strains showed an increase in survival. Mutant (BSe50/20/1) showed a high survival rate, with a 95.64% survival rate. Under different hydrogen peroxide concentrations (0.75 mM H_2_O_2_ and 2.25 mM H_2_O_2_), the mutant (BSe50/20/1) maintained a high survival rate.

#### Tolerance to Bile Salts

The difference in viable cell counts after 8 h of incubation was used to estimate the survival rate of the selected strains, as indicated in Fig. 1c. All strains demonstrated an improvement in survival when exposed to 0.25% bile-salt. Mutant (BSe50/20/1) had a high survival rate of 95.64%.

#### Lysozyme Response Assay

The resistance of the four strains to varied amounts of lysozyme ranged from a minimum value of 53.85% to a maximum value of 81.72%. After 120 min of treatment, all mutant strains demonstrated strong lysozyme resistance, in comparison with the parental strains, as shown in Fig. 1d. In general, the resistance of the four strains to 100 mg/L lysozyme was higher than the resistance of the four strains to 200 mg/L lysozyme.

#### Total Antioxidant Activity (TAA)

The antioxidant capabilities of the selected strains were assessed by inhibiting ascorbic acid autooxidation. The antioxidant activity percentages ranged from 14.4 (ProBl) to 28.4 (PSe40/60/1). As indicated in Fig. 1e, all mutant strains had significantly higher antioxidant activity percentages than the parental strains did.

#### Antibiotic Response of the Mutants and Wild-Type Probiotics Strains

The antibiotic responses of probiotic cultures ranged from sensitive (S) to resistant (R) to seven tested antibiotics, as shown in Table 5. Probiotic (Pro1) was sensitive to all the seven tested antibiotics with high sensitivity to chloramphenicol. Probiotic mutant PSe40/60/1 was resistant to chloramphenicol, kanamycin, and streptomycin, but susceptible to the other antibiotics, with a high susceptibility to lincomycin. Probiotic (ProBl) was resistant to kanamycin and lincomycin, but susceptible to the other antibiotics, with a high sensitivity to ampicillin. Probiotic mutant BSe50/20/1 was resistant to erythromycin, lincomycin, and streptomycin, but susceptible to the other antibiotics, with a high susceptibility to ampicillin.

**Table 5:** Show antibiotic response of the two mutants and wild types of probiotics strains under different antibiotic discs after 24 h

Probiotic code	Antibiotic disc						
	*AMP(^+^10)	C(30)	ERY(15)	K(30)	L(2)	S(10)	TOB(30)
Pro1	**16	18	17	13	11	13	16
PSe40/60/1	15	R	14	R	16	R	7
ProBl	22	20	14	R	R	12	8
BSe50/20/1	19	13	R	8	R	R	11

^*^AMP ampicillin, *C* chloramphenicol, *ERY* erythromycin, *K* kanamycin, *L* lincomycin, *S* streptomycin, *TOB* tobramycin. + Concentration (µg/disc). *R* resisted (no clear zone)

^**^Means of diameter of inhibition zones

#### Mice Body Weight from Zero Time to 45 Days

**Fig. 2:**
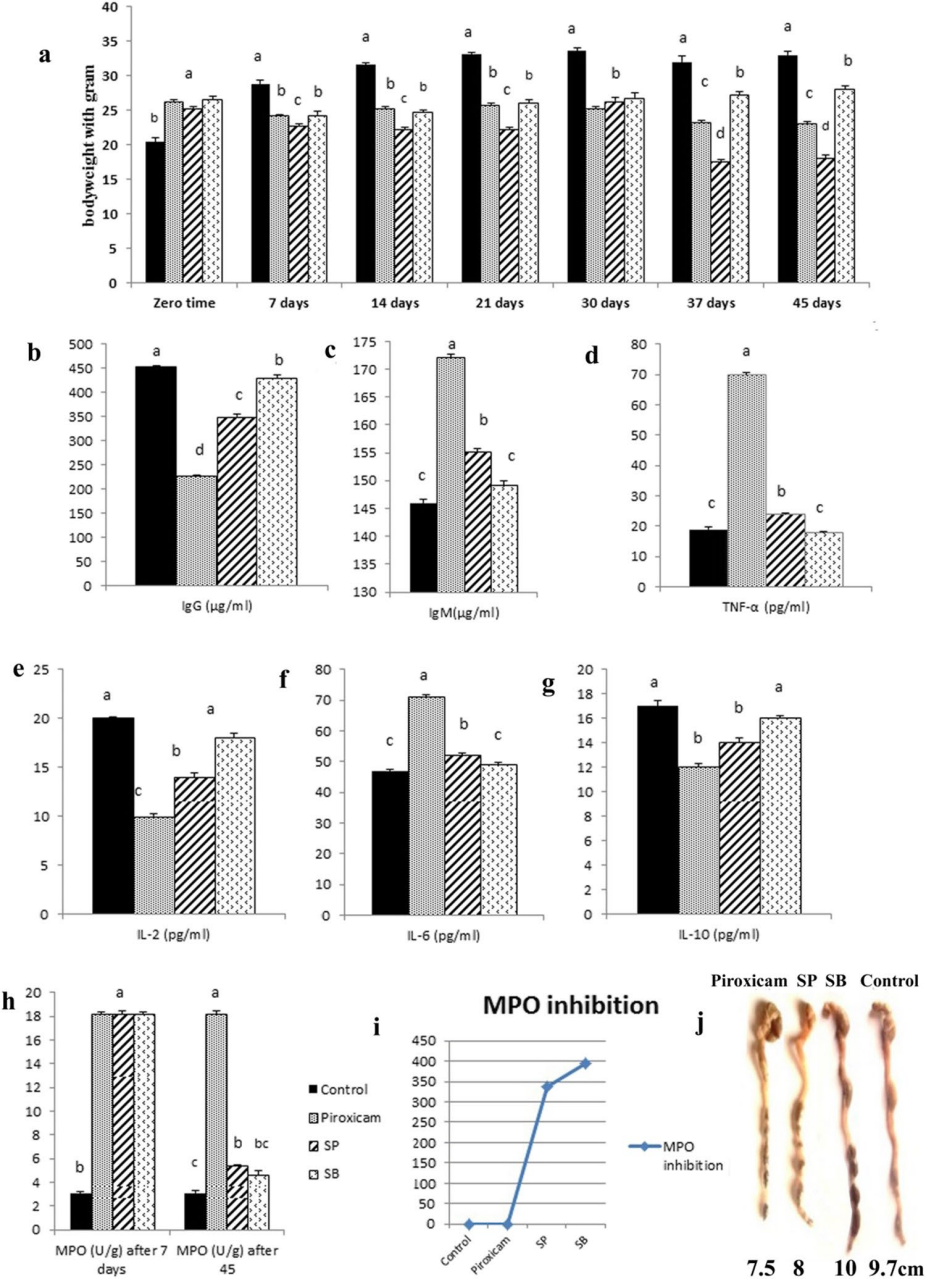
**a** Bodyweight of mice; **b**–**g** ELISA analysis of IgG, IgM, TNF-α, IL-2, IL-6, and IL-10; **h**, **i** biochemical analysis of Myeloperoxidase activity; and **j** colon morphology and length after probiotic mutants’ treatments

Although the bodyweight of control mice was the lowest when compared with the other groups at the zero time, it became the highest after 7 days, when compared with other groups treated with the Piroxicam drug. After 2 weeks of the experiment, SP and SB groups were treated with PSe40/60/1 and BSe50/20/1, respectively, alongside the Piroxicam. On day 21, bodyweight increased in SB group and decreased in SP group, compared to their original values before treatment with the mutant strains. In comparison to the other groups, the SP group reported the lowest weight during the experiment period (Fig. 2a).

#### Colon Morphology and Length After Treatments of Probiotic Mutants

As demonstrated in Fig. (2j), the Piroxicam drug substantially reduced colon length when compared to the control group, whereas PSe40/60/1 mutant significantly reduced colon length when compared to the BSe50/20/1 mutant group. The colonic length was somewhat recovered after treatment with BSe50/20/1 mutant, indicating that BSe50/20/1 could prevent colon shortening.

### Biochemical Analysis

#### MPO Activity

The level of MPO activity was low in the negative control group, high in the positive control group, and lower in SP and SB treatment groups than in the positive control group. After 45 days, the SB treatment had lower MPO activity than the other treatments (Fig. 2h and i).

#### Serum Inflammatory Immunoglobulin and Cytokines

The results of ELISA analysis in serum showed that IgG, IL-2, and IL-10 levels significantly reduced in the Piroxicam group compared with the other group. IgM, IL-6, and TNF-α levels significantly increased in Piroxicam group compared with the other group. No significant differences were observed between SB group and control group in IgM, IL-6, IL-10, and TNF-α level (Fig. 2b–g).

#### Hematological Parameters After Probiotic Mutants’ Treatments

Results of CBC showed that the measurements of the red cells, hemoglobin, neutrophil, monocyte, eosinophil, and basophil in blood did not record any significant differences for all groups, whereas the white blood cells increased significantly in the Piroxicam group, compared with the other group. Platelets decreased significantly in the Piroxicam group, compared with the other group (Table 6).

**Table 6:** Hematological parameters after probiotic mutants’ treatments in comparison of different controls

CBC	Control	Piroxicam	SP	SB	*P*-value
RBCs (million/mm^3^)	5.2 ± 0.1	4.5 ± 0.2	4.7 ± 0.1	4.3 ± 0.3	0.07
Hb (g%)	14 ± 0.2	15.5 ± 0.2	14.3 ± 0.3	13.5 ± 0.5	0.057
WBC (thousand /mm^3^)	5400^c^ ± 0.9	6600^a^ ± 0.9	5000^d^ ± 0.8	5700^b^ ± 0.8	0.03
Platelets (thousand /mm^3^)	850000^a^ ± 1.2	600000^d^ ± 1	700000^c^ ± 1.3	720000^b^ ± 1	0.01
Neutrophil (10^3^/µl)	13^cs^ ± 0.1	26^a^ ± 0.2	23^b^ ± 0.3	23^b^ ± 0.2	0.04
Monocyte (10^3^/µL)	2	1	0	1	––-
Eosinophil (10^3^/µL)	1	0	0	1	––-
Basophil (10^3^/µL)	0	0	0	0	––-

### Histopathology Results

**Fig. 3:**
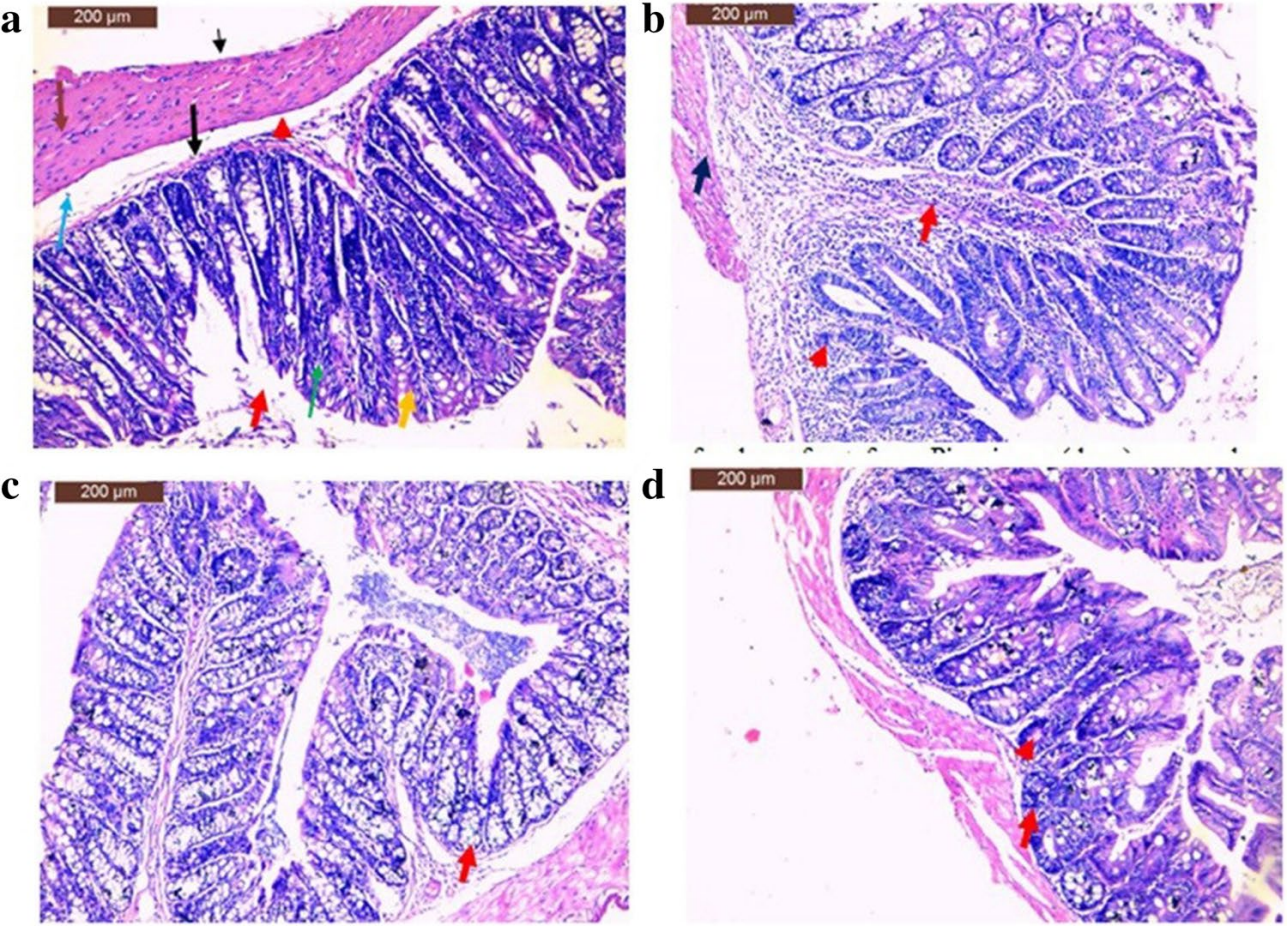
**a** A section of colon of mice from normal negative control showing the normal histological features of the simple columnar epithelium (red arrow) that has abundant goblet cells (yellow arrow) interspersed with absorptive cells (green arrow) and large crypts of Lieberkühn (arrow). Note the absence any glands in the submucosa (red arrow head), **b** a section of colon from positive control group showing marked massive inflammatory cells infiltrates in the lamina propria (red arrow) with complete crypt destruction and a single regeneratory layer of epithelial cells covering the lamina propria (arrowhead). Note the prominent inflammation within the wall of the colon (blue arrow), **c** a section of colon from SP group showing mild reduced in inflammatory cells infiltrates. Note crypt appeared more or less like normal (arrow), **d** a section of colon from SB group showing markedly reduced in inflammatory cells infiltrates. Note the prominent lamina propria infiltration as well as infiltration of mononuclear cells into the submucosa (arrow). Partial loss of crypts (arrowhead) was found (hematoxylin and eosin stain, scale bar 200 µm)

The histopathological studies of colon sections from mice in the control group showed a normal histological structure of the simple columnar epithelium that had abundant goblet cells, interspersed with absorptive cells and large crypts of Lieberkühn. No glands in the submucosa were found (Fig. 3a). Colon sections of the Piroxicam group exhibited massive inflammatory cells that infiltrated the lamina propria associated with complete crypt destruction and the single regenerative layer of epithelial cells covering the lamina propria. The prominent inflammation within the colon wall was noted (Fig. 3b). The examination of colon sections of SP group showed mild reduction in the infiltrating inflammatory cells. The crypt appeared more or less like normal (Fig. 3c). The microscopic investigation of the colon of SB group showed a marked reduction in the infiltrating inflammatory cells. Prominent lamina propria infiltrations, as well as the infiltration of mononuclear cells into the submucosa, and partial loss of crypts were detected (Fig. 3d).

**Fig. 4:**
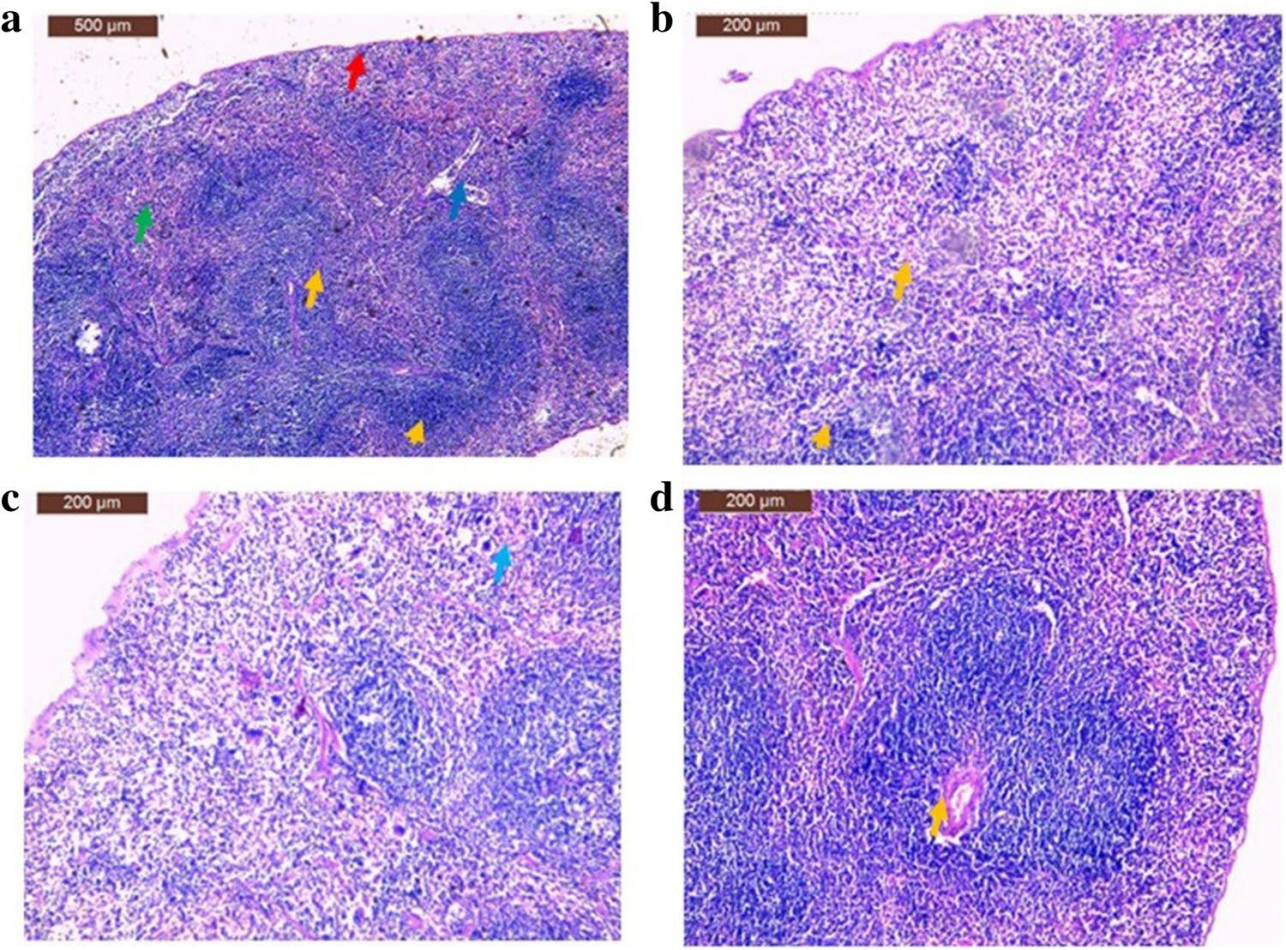
**a** A section of spleen of mice from normal negative control group showing capsule (red arrow) and melanomacrophages (arrowhead), white pulp (yellow arrow), red pulp (green arrow), and spleen trabecula (blue arrow), **b** a section of spleen from positive control group show hemorrhage beneath the capsule hemorrhage in splenic parenchyma (arrow) and an increase in the number of melanomacrophages (arrowhead), **c** a section of spleen from SP group showing hypertrophic plasma cells (arrow), and **d** a section of spleen from SB group showing the main structure exhibit nearly normal form (hematoxylin and eosin stain, scale bar 200 µm)

In mice of the control group, the main components of the spleen were white and red pulps encircled with a capsule made up of connective tissue and melanomacrophages (Fig. 4a). In the Piroxicam group, the Piroxicam drug caused hemorrhage beneath the capsule in the splenic parenchyma and an increase in the number of melanomacrophages (Fig. 4b). In the case of spleen sections of SP group, hypertrophic plasma cells were presented (Fig. 4c). On the other hand, the spleen of SB group showed that the main structure exhibited nearly normal form (Fig. 4d).

### Gene Expression

**Fig. 5:**
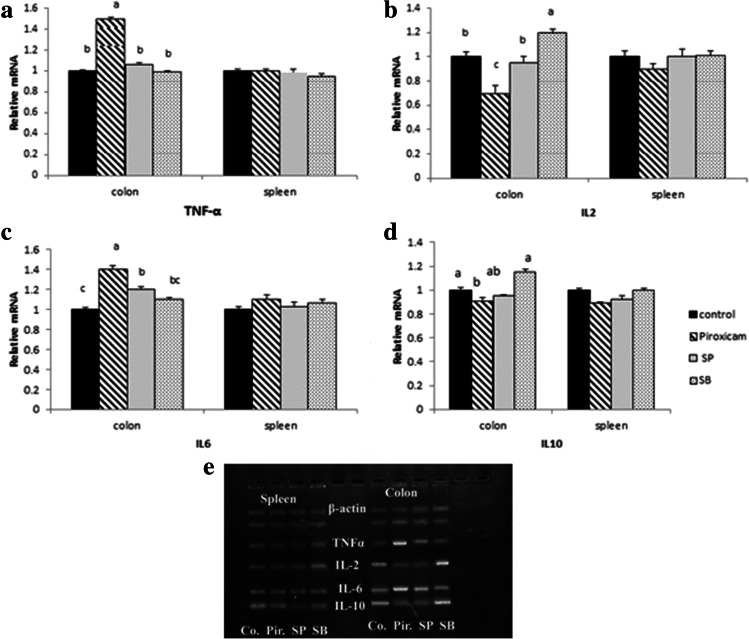
Expression of TNF, IL-2, IL-6, and IL-10 genes in colon and spleen

Expression of TNF-α and IL-6 genes was significantly upregulated in colon of the Piroxicam group compared with the control group. Expression of IL-6 gene was upregulated in colon of the SP group compared with the control group, while non-significant differences were observed between SB and control groups in expression of TNF-α and IL-6 genes. Expression of IL-2 and IL-10 were significantly downregulated in colon of the Piroxicam group while significantly upregulated in colon of the SB group when they were compared to the control group. Also, there are no differences between all groups in expression of TNF-α, IL-2, IL-6, and IL-10 genes in spleen (Fig. 5a–d). The density of β actin bands was equal between all groups in colon and spleen. This density is insignificantly differentiated in the other tested genes in spleen while significantly differentiated in colon. These significant differences in band density are appropriate to upregulation of TNF-α and IL-6 in the Piroxicam group and IL-2 and IL-10 in SB group compared with control group (Fig. 5e).

## Discussion

Several studies have shown that gut bacteria play an essential role in the development of UC [24] and that probiotic supplementation is beneficial for UC [25]. Since the majority of UC damage occurs in the colon or rectum and because various bacteria have distinct secretory activities or metabolism in the intestinal tract, a local bacteria complement is more useful for UC. Clearly effective probiotics should be chosen for a comparative investigation of their impact on experimental colitis because probiotics have good permanent planting potential. Several studies on *Escherichia coli* Nissle, *Lactobacillus casei*, *Bifidobacterium lactis*, *Lactobacillus acidophilus*, and other probiotics have concluded that they can be utilized to treat IBD [6, 26]. The findings of the present study reveal that after mutagenic treatment of *L. plantarum* Pro1 and *B. longum* ProBl with 200 mM EMS for 60, 20, and 20 min, large amounts of selenium-resistant fast-growing mutants were obtained. The two probiotic mutant strains (PSe40/60/1 and BSe50/20/1) were chosen based on their ability to absorb a significant quantity of selenium, which is a crucial attribute of mutants to be utilized as probiotic strains. The above results are in agreement with those by Tünde et al. [27]. Results demonstrate that mutants of *Enterococcus faecium* W54, *Lactococcus lactis* ssp. *lactis* R703, *Bifidobacterium animalis* ssp. *lactis* BB12, and *Lactobacillus casei* 431resistant to high selenite concentrations were selected, marking that the stress tolerance of the mutant strains differed from that of the parental cultures. The differences in thermal tolerance were particularly noticeable, as the optimum development of the mutant strains was pushed to higher temperatures than in the case of the parental strains. Salt and pH resistance were also crucial in the *L. casei* 431 mutant. Following mutagenesis, the mutant of *L. casei* 431 acquired approximately 10 times more selenium uptake.

Probiotic properties, such as bile resistance and acid tolerance, were tested, since survival under the environmental conditions of the gastrointestinal system is a crucial characteristic feature of the isolate to be employed as a probiotic. In vitro, the present research found that the used mutant of selenide-enriched probiotics can survive pH 2 and grow in 0.25% bile salts, indicating that they meet the fundamental probiotic requirements. Bile salt tolerance varies greatly among LAB species and even within strains. The activity of the enzyme bile salt hydrolase (BSH), responsible for the breakdown of bile salts, thereby the reduction of their toxic impact, can be related to LAB resistance to bile salt [28]. The results of this study are similar to those by Fossi et al. [29], who reviewed the survival rate of LAB at pH 3. In general, LAB can elicit an acid tolerance response (ATR) to acidic stress [30], leading to pH homeostasis and repair, which eventually make species resistant to low pH. In the course of the present study, both mutants showed greater antioxidant activity percentages and resistance to pH 2, 2.25 mM H_2_O_2_, 0.5% bile salt, and 200 mg/L of lysozyme than the original strains. Thus, the two probiotic mutant strains were required to analyze the therapeutic effects on experimental colitis and to demonstrate that probiotics may be used to treat colitis. By the end of the experiment, weight loss had halted, and weight increase even occurred. These findings corroborate the previously published findings [31]. MPO activity considerably declined in all probiotic treatment groups. In addition, following the treatment with the Piroxicam drug, all estimates of the immune proteins and cytokines under study were altered and then reverted to normal or near-normal levels post-treatment with probiotics. These results are similar to those by Shahbazi et al. [32] and Zhang et al. [33].

Moreover, the estimates changed for all treatments at the level of the blood picture, as red blood cells decreased, due to bleeding and colon inflammation, whereas white blood cells increased after treatment with the drug, due to colon inflammation, and the immune system’s attempt to overcome problems associated with drug administration, hence reverted to the values post probiotic treatments. There was also a shortage of platelets in all treatments compared to the control, while platelets increased after treatments. These results are in agreement with those by Kilany et al. [34] and Tarabees et al. [35]. The histological examinations of the colon and spleen demonstrated that the Piroxicam drug had a severe effect on them. On the other hand, the used mutant strains of probiotics that were supplemented with selenium restored the colon and spleen to normal or near-normal condition, since SB treatment was superior to SP treatment. These results are in agreement with those by Chorawala et al. [36] and Esposito et al. [37].

The expression of TNF-α, Il-2, and Il-6 genes significantly increased in the colon of the Piroxicam group, compared with the other groups, while the expression of IL-10 gene recorded the lowest value in the Piroxicam group, compared with the other groups. In the spleen, non-significant variations in the expression of TNF, Il-2, Il-6, and Il-10 genes were observed in all groups. The Il-6 gene considerably increased in the colon of the SP group, compared with the control group, whereas the SB and control groups showed no significant alterations. Previous studies concluded that specific probiotic strains could activate DCs, which then transfer antigens to local lymph nodes, releasing IL-10 and IL-12 in the process. According to these studies, the immunological modulation caused by probiotic bacteria may be related to the production of an anti-inflammatory cytokine in the gut. However, the exact molecular interactions between probiotics and their hosts are unknown. *Lactobacillus* strains may influence immune cell cytokine production, while *Bifidobacterium* induce tolerance acquisition [32, 33]. The differences in the regulatory activities of each probiotic strain are due to strain properties, the spectrum of mediators released, and the various pathways activated simultaneously. Choi et al. [38] found that oral treatment of *L. plantarum* strain CAU1055 substantially reduced the levels of inducible NOS, COX-2, TNF-α, and IL-6. A strain of *L. plantarum* C88 appeared to protect the mice against liver damage by downregulating the levels of IL-8, IL-1, IL-6, IFN-, and TNF- and inhibiting the NF-B signaling pathways, hence lowering TLR2 and TLR4 expression [39]

## Conclusion

The present evaluation of the critical role of the two selenium-enriched PSe40/60/1 and BSe50/20/1mutant strains as anti-inflammatory and immunomodulatory supplements is the first study in this field. *Lactobacillus plantarum* Pro1 *and Bifidobacterium longum* ProB1 were used to induce several mutant strains. PSe40/60/1 and BSe50/20/1 mutant strains were selected from 21 mutant strains and loaded with selenium. The BSe50/20/1 mutant strain caused the reduction of IgM, IL-6, and TNF-α levels and the increase of IgG, IL-2, and IL-10 in serum. It also caused a marked reduction of myeloperoxidase activity, in addition to colon inflammation induced by the Piroxicam drug, while it exhibited nearly normal form for the spleen. These beneficial effects were accompanied by an expression of TNF-α, IL-2, IL-6, and IL-10 genes in normal levels.

## Data Availability

The datasets used and/or analyzed during the current study are available from the corresponding author on reasonable request.
